# Efficiency of Soybean Products in Broiler Chicken Nutrition

**DOI:** 10.3390/ani12030294

**Published:** 2022-01-25

**Authors:** Alina Janocha, Anna Milczarek, Daria Pietrusiak, Kamil Łaski, Mohamed Saleh

**Affiliations:** 1 Institute of Animal Science and Fisheries, Faculty of Agrobioengineering and Animal Husbandry, Siedlce University of Natural Sciences and Humanities, Bolesława Prusa 14, 08-110 Siedlce, Poland; alina.janocha@uph.edu.pl (A.J.); dp335@stud.uph.edu.pl (D.P.); kamillaski@gmail.com (K.Ł.); 2 Department of Poultry, Faculty of Agriculture Production, Sohag University, Street Nasser City, Sohag 82524, Egypt; moh_sa_al@yahoo.com

**Keywords:** soybean meal, soybean expeller cake, extruded full-fat soybean, nutrition, broiler chicken

## Abstract

**Simple Summary:**

The studies show how replacing soybean meal with soybean expeller cake or extruded full-fat soybean in feed rations for broiler chickens affects rearing results (weight gain, intake and conversion of feed), carcass composition (dressing percentage, musculature, fattens) and meat quality (physical properties: pH, colour, water holding capacity; the chemical composition: basic components, fatty acids and organoleptic value: flavour, tenderness, palatability and juiciness). Based on the results the use of soybean expeller cake in broiler chicken starter/grower diets can be recommended as it allowed for obtaining the best production and slaughter results. However, from the point of view of the human diet, extruded full-fat soybean should be suggested since it best modified the lipid fraction of muscles.

**Abstract:**

The study aimed to determine the overall effect of replacing soybean meal completely with soybean expeller cake or extruded full-fat soybean in feed rations for broiler chickens on their carcass composition and meat quality. The experiment involved one hundred and twenty Ross 308 broiler chickens randomly allocated to three equinumerous groups (SBM, SEC, EFS). Each group was divided into five subgroups—each consisting of eight birds of both sexes (1:1). From Day 1 to Day 21 of life the birds were fed with loose starter rations, and from Day 22 to Day 42 they were fed grower rations based on a wheat meal, protein products and mineral and vitamin admixtures. The experimental factor was a protein raw material comprising: SBM group—soybean meal from GM (genetically modified) seeds, SEC—soybean expeller cake from n-GM seeds, and EFS—extruded full-fat soybean from n-GM seeds. The experimental diets were isocaloric and isonitrogenous. It was demonstrated that replacing soybean meal with SEC or EFS in feed rations for broiler chickens led to a significant (*p* < 0.05) weight gain on Day 42 of rearing by 4.57% and 2.88%, respectively. The chickens fed diets with EFS had worse (more than 4.14%) feed conversion rate (FCR) in comparison to the others (*p* < 0.05). Broiler chickens from the SBM and SEC groups showed a higher share of breast and leg muscles (by 4.74% and 7.54%) and a lower share of abdominal fat (by 31.1%) and skin with subcutaneous fat (by 18.8% and 13.4%) in comparison with birds from the EFS group (*p* < 0.05). The highest content of intramuscular fat with the best fatty acids profile was determined in the muscles of birds fed with diets containing EFS, while the muscles of chickens receiving SEC scored the highest. The results provide grounds for recommending SEC in broiler chicken nutrition as it allowed for obtaining the best production and slaughter results, whereas—from the point of view of the human diet—EFS should be recommended since it best modified the lipid fraction of muscles.

## 1. Introduction

The production of poultry, including broiler chickens, is a major area of animal production. Challenging the future growth of it are several factors such as poultry immunity and health, and balanced feeding rations. One of those is a lasting pandemic. It is essential to know that broiler chickens are not susceptible to infection by the SARS-CoV-2 virus. Nevertheless, the COVID-19 pandemic will affect poultry consumption and the economics of poultry farming [1,2].

It requires continuing supplies of protein components covering the requirement of the animal feed industry. Imported soybean meal is a commonly used protein component in poultry feed rations. Nearly all (ca. 95%) varieties are genetically modified [3,4], which—considering the prospect of introducing a ban on using GM materials after 1 January 2024—can be a material problem in the production of poultry. Annually, EU member states import about 36 million tonnes of soybean products, including about 12 million tonnes of seeds and 19 million tonnes of soybean meal. The Institute of Agricultural and Food Economics estimates that in Poland soybean meal covers about 62% of the feed protein requirement and 23% of protein is sourced from rapeseed components, about 7.5% from sunflower seed meal, while legume seed protein accounts for only 6.5%. Every year Poland imports about 2.5 million tonnes of soybean meal [5].

An alternative to soybean meal from GM varieties can be soybean products grown in European countries. Feed producers have been improving their technical potential of heating and pressure-thermal processing of imported and domestic soybean seeds [6,7,8,9,10,11]. Processing of soybean seeds for consumption results in multiple products that can be used in animal nutrition, i.e., soybean meal (from dehulled seeds, with different particle size, extracted, expelled), soybean expeller cake, soybean oil, extruded full-fat soybean, isolated soybean protein, and soybean hull [1,8,9,11,12]. All these products differ in terms of the content of nutrients and thus can be fed to different species of animals [11,13,14]. The use of soybean products is limited by the presence of anti-nutrients—mainly inhibitors of proteolytic enzymes such as trypsin and chymotrypsin [15,16,17]. Studies are not unequivocal about the outcomes of broiler chicken rearing [6,7,8,18,19,20,21]. Mirghelenj et al. [21] showed a decreased bodyweight of chickens fed rations with a higher share of extruded full-fat soybean. In turn, Milczarek et al. [6] showed that introducing extruded full-fat soybean into feed rations as a partial substitute of soybean meals allowed for achieving a similar bodyweight of chickens at a similar feed consumption level. Śliwa and Brzóska [7], analysing the effect of replacing soybean meals with extruded soybean expeller cake in the feed ration, showed that as the share of expeller cake increased in the diet of birds, their bodyweight decreased and the feed conversion rate increased. In turn, Powell et al. [19] did not note any significant difference in the carcass weight of slaughtered birds and in feed conversion rate after introducing expeller-extruded soybean meal as the only source of protein.

The aim of this study was to determine the effect of soybean expeller cake and extruded full-fat soybean added in place of soybean meal to broiler chicken feed on performance results, carcass composition and meat quality.

## 2. Materials and Methods

### 2.1. Experiment Design

The feeding experiment involved one hundred and twenty Ross 308 broiler chickens randomly allocated to three equinumerous groups (SBM, SEC, EFS). Each group was divided into five subgroups—each consisting of 8 birds of both sexes (50% cocks and 50% hens). The chickens were reared over a 42-day cycle under standard microclimate conditions with unlimited access to water (nipple drinkers) and feed (feeder). An ad libitum feeding scheme was used. Throughout the rearing period the birds were exposed to 24-h electric lighting. In the first experimental week the ambient temperature was 32 °C, and was then decreased each week (every seven days) by 1–2 °C until it reached 21–23 °C in the last rearing week. In the first rearing period, that is, until Day 21, the birds were fed complete bulk starter feed rations and from Day 22 to 42—with grower feed rations. All feed rations were produced from wheat meal and protein raw materials with mineral and vitamin admixtures. The experimental factor was a protein raw material comprising: SBM group—soybean meal from GM seeds, SEC—soybean expeller cake from non-GM seeds, and EFS—extruded full-fat soybean from non-GM seeds. Testing of non-GM soybean products was properly certified by J.S. Hamilton Poland S.A. Gdynia—appraisals and laboratory tests (Accredited method—PCA Accreditation Certificate No. AB 079).

The nutritional value of feed rations was assessed based on the content of feed raw material ingredients (Table 1) according to dietary recommendations [22]. The ingredients and nutritional value of diets are listed in Table 2.

During the growth experiment the birds’ bodyweight was controlled on Day 1, 21 and 42 along with the intake of feed in respective rearing periods. The results were used to calculate weight gain and feed conversion rate (FCR).

On the 42nd day of the birds’ life, ten birds with a body weight representative of a specific group were selected from each group and slaughtered by decapitation, earlier chickens were mechanical method stunned. Fifteen minutes after the slaughter the reaction (pH_15_) was measured in their breast muscles (*M. pectoralis maior*) and thigh muscles (*M. iliotibialis*). Next, the carcasses were cooled over 24 h at a temperature of 4 °C and afterward the reaction (pH_24_) of the muscles was measured again. In order to calculate the dressing percentage, the weight of cooled carcasses was determined and they were subject to simplified dissection analysis using a procedure described by Ziołecki and Doruchowski [23]. During dissection samples of breast and leg muscles were taken for evaluating their physico–chemical and sensory characteristics.

### 2.2. Chemical Composition Evaluation of Soybean Products (n = 3) and Muscles (n = 10)

The dry matter, total ash, crude protein and crude fat contents were described by the AOAC [24] according method number: dry matter (930.15), total ash (942.05), crude protein (990.03), crude fat (991.36). The gross energy of soybean products was determined using an Oxygen Bomb Calorimeter [25]. The number of nitrogen-free extractives (NFE) was calculated from the formula:

NFE = dry matter − (crude protein + total ash + crude fat + crude fiber)

In addition, anti-trypsin activity was determined in soybean raw materials using a method designed by Smith et al. [26], involving spectrophotometric assay of absorption of the products of casein breakdown by trypsin in the presence of an inhibitor. Tannins were determined in processed soybean seeds according to BN-90/79160-62 [27]. The method consisted of extracting tannins using a mixture of ethyl alcohol, glycerine and water, creating a coloured complex with phosphomolybdenum–phosphowolfram reagent and measuring absorption of the coloured solution at wavelength 700 nm.

The fatty acid profile in muscles was determined by gas chromatography [28]. Fatty acid analysis was made with gas chromatography (GC) using gas chromatograph (GCMS-QP210 Ultra, Shimadzu, Kyoto, Japan) with capillary column and flame-ionisation detection and helium as the carrier gas. The initial oven temperature was 140 °C for 1 min, thereafter increased by 20 °C/min to 200 °C and held for 20 min and increased by 5 °C/min to 220 °C held for 25 min. The injector was heated to 250 °C and the detector to 270 °C. FAME standards (Supelco 37 Component FAME Mix, Bellefonte, PA, USA) were used to identify the fatty acids present in the samples. Based on the percentage (% of the total) of fatty acids, we calculated the neutral and hypocholesterolemic fatty acids (DFA) and hyperholesterolemic fatty acids (OFA).

### 2.3. Physical Properties Evaluation of Muscles (n = 10)

The concentration of hydrogen ions (pH_15_ and pH_24_) in pectoralis major and iliotibial muscles was measured using a Testo 205 pH-meter with a dagger electrode.

Water losses, expressed as water holding capacity (WHC) was determined by Grau and Hamm’s filter-paper press method described by Jurczak [29] based on the surface of meat juice on the filter paper.

The colour of breast muscles was determined using a Minolta Chroma Metters CR 300 (Konica Minolta, Osaka, Japan) instrument according to the L, a*, b* system [30]. Two illuminant/observer combinations were applied, i.e., illuminant C (average daylight) and standard observer 2° as well as illuminant D65 (day light) and standard observer 10°, recommended for measurements of meat colour [31]. In the used measuring system L denotes psychometric colour saturation that is a spatial vector. On the other hand, a* and b* are trichromatic coordinates, where a* as a positive value corresponds to red, and as a negative value—green; in turn, positive b* corresponds to yellow, and negative b*—blue. The colour parameters a* and b* were used to calculate chroma (C) and hue (H) with formulas used by [32].

### 2.4. Organoleptic Properties of Muscles (n = 8)

The organoleptic properties of breast and thigh muscles were evaluated on a five-point scale after cooking in a 0.80% NaCl solution up to a temperature of 80°C in the geometric centre of the sample. The meat to water ratio was 1:2. The flavour of muscles in terms of palatability, flavour, juiciness and tenderness was evaluated by a group of eight trained people [33,34].

### 2.5. Statistical Analysis

The results were elaborated by statistical methods using one-way analysis of variance (ANOVA), according to the following mathematical model:

*Y_ik_* = *μ* + *a_i_* + *e_ik_*

where:

*Y_ik_*—value of the analysed feature,
*μ*—total mean value,
*a_i_*—effect of the experimental factor,
*e_ik_*—error.

The significance of differences between mean values was verified using Tukey’s test at the significance level α < 0.05. The results were elaborated using STATISTICA PL 13.1 (StatSoft, Inc., Krakow, Poland) software [35].

## 3. Results

The nutritional value of protein-rich raw materials used in the growing experiment involving broiler chickens is presented in Table 1. Among the evaluated soybean raw materials, the highest content of total protein was determined in soybean meal (452 g·kg^−1^). It was slightly lower in soybean expeller cake (443.6 g·kg^−1^), and the lowest in extruded full-fat soybean (349.5 g·kg^−1^). In turn, the highest content of crude fat (218 g·kg^−1^) was found in extruded full-fat soybean and the lowest (20.7 g·kg^−1^)—in soybean meal, which was reflected in the gross energy level of the evaluated raw materials. In comparison to soybean meal, soybean expeller cake and extruded full-fat soybean contained more (by 4.50 g·kg^−1^ and 7.48 g·kg^−1^, respectively) trypsin inhibitors and less (by 12.0 g·kg^−1^ and 9.82 g·kg^−1^, respectively) tannins.

After the starter feeding period (21 days of life), chickens receiving diets containing soybean expeller cake showed a significantly (*p* < 0.05) higher (by about 5.41 and 6.29%) bodyweight than birds fed with rations containing soybean meal or extruded full-fat soybean as a protein raw material (Table 3).

Replacing soybean meal with SEC or EFS in feed rations for broiler chickens led to a significant (*p* < 0.05) weight gain on Day 42 of rearing. The highest weight gain in both rearing periods was observed for chickens fed with diets containing SEC and statistically significant differences were found for birds from the SBM group. The nutrition scheme used significantly modified the conversion of feed at respective rearing periods. The lowest FCR was characteristic of birds receiving feed rations containing SEC and the highest—of chickens fed diets with extruded full-fat soybean (*p* < 0.05).

Replacing soybean meal with SEC or EFS in feed rations significantly (*p* < 0.05) increased pre-slaughter bodyweight (Table 4).

The nutrition scheme had no impact on the dressing percentage of chickens but did modify their musculature and fattiness. Broiler chickens from SBM and SEC groups showed a higher share of total muscles (by 4.74% and 7.54%) and a lower share of abdominal fat (by 31.1%) and skin with subcutaneous fat (by 18.8% and 13.4%) in comparison with birds from the EFS group (*p* < 0.05). The lowest share of giblets (heart, liver, gizzard) in bodyweight was recorded for chickens receiving feed rations with soybean expeller cake as the only source of protein (*p* < 0.05).

The protein raw material used in broiler chicken feed rations significantly altered the content of dry mass in breast muscles and crude fat in both muscles (Table 5).

The lowest content of crude fat was determined in both breast and leg muscles of birds from the SEC group in comparison to the SBM group and the EFS group. The differences were confirmed to be statistically significant between SEC and EFS groups only (*p* < 0.05).

Considering the dietary value of meat, careful attention is paid not only to the amount of intramuscular fat but primarily to its fatty acids profile. The composition and share of fatty acids in the lipids of breast and leg muscles are presented in Table 6.

Extruded full-fat soybean used as the only protein component in feed rations for broiler chickens contributed to a significant (*p* < 0.05) decrease in the content of palmitic acid (C16:0), stearic acid (C18:0), and—consequently—saturated fatty acids (SFA) and hypercholesterolemic fatty acids (OFA) in both evaluated muscles in comparison to muscles of birds from other groups. A significantly (*p* < 0.05) lowest content of oleic acid (C18:1) and monounsaturated fatty acids (MUFA) and highest content (*p* < 0.05) of linoleic acid (C 18:2) and linolenic acid (C 18:3) and polyunsaturated fatty acids (PUFA) was noted in the muscles of birds from the EFS group in relation to SBM and SEC groups.

The type of soybean raw materials used in feed rations had no statistically significant (*p* > 0.05) effect on the reaction of breast muscles measured 15 min after slaughter and then after 24 h of cooling (Table 7). The pH in the thigh muscles was slightly different. The muscles of all chickens 15 min after slaughter showed a similar reaction (5.88–5.90), while after 24 h of cooling the strongest (*p* < 0.05) acid reaction was noted in the muscles of birds fed with diets containing extruded full-fat soybean.

The nutrition scheme was not found to have an effect on the lightness (L*) of the breast and thigh muscles colour. The breast muscles of chickens receiving feed rations with soybean expeller cake showed a nearly two times higher red saturation (a*), yellow saturation (b*) and chroma (C*) than the muscles of birds from other groups (*p* < 0.05). In turn, in the thigh muscles, the highest red saturation (a*), and the lowest yellow saturation (b*) were observed for the muscles of birds fed with diets containing extruded full-fat soybean as the only source of protein (*p* < 0.05). The breast and thigh muscles of chickens receiving feed mixtures containing soybean expeller cake (*p* < 0.05) showed high water holding capacity (WHC) in comparison to the muscles of birds from the SBM and EFS groups.

**Figure 1:**
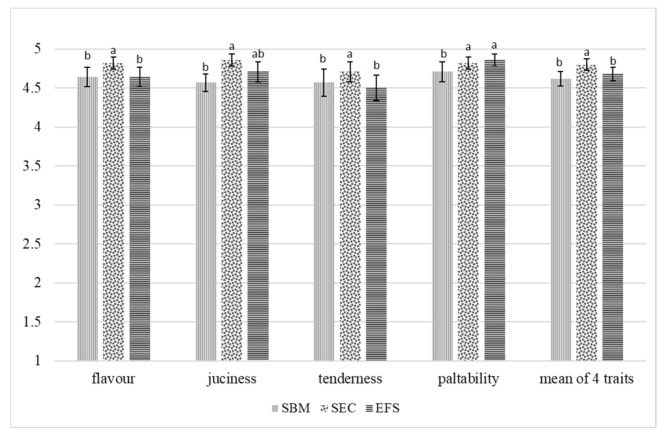
Sensory evaluation of breast muscles (point). a,b—means with different superscripts within a row are significantly different at *p* < 0.05.

**Figure 2:**
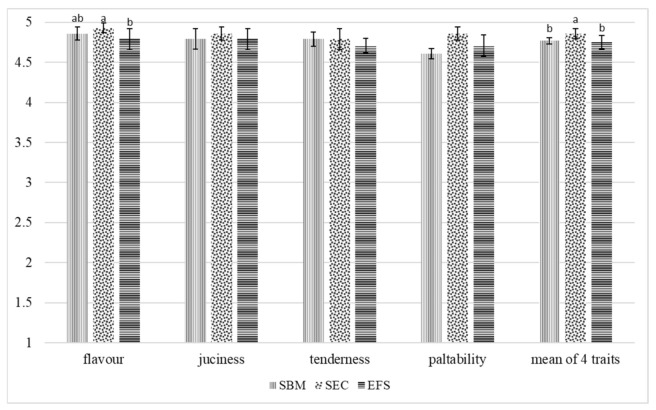
Sensory evaluation of thigh muscles (point). a,b—means with different superscripts within a row are significantly different at *p* < 0.05.

Replacing soybean meal with soybean expeller cake or extruded full-fat soybean in feed rations for broiler chickens affected the sensory traits of muscles (Figure 1 and Figure 2).

The breast muscles of chickens fed with rations containing soybean expeller cake had the highest score for all flavour traits (smell, juiciness, tenderness and palatability) than the breast muscles of birds receiving feed rations with soybean meal or extruded full-fat soybean. Similarly, as regards thigh muscles, those of chickens from the SEC group scored highest, with a statistically confirmed difference recorded for smell only. Considering the mean scores for all the evaluated sensory traits, both types of muscles of chickens from the SEC group scored significantly (*p* < 0.05) better than those of birds from the SBM and EFS groups.

## 4. Discussion

Total protein content determined in soybean meal and extruded full-fat soybean was close to amounts (465.8 g·kg^−1^ in soybean meal and 337.9 g·kg^−1^ in extruded full-fat soybean) reported by Milczarek et al. [6]. Similarly, Grela and Czech [5] showed that extruded full-fat non-GM soybean seeds contained from 320 to 360 g·kg^−1^ total protein. Bandegan et al. [36] found a higher share (44.6–46.7%) of this ingredient in dry-extruded soybean meal. The content of protein similar to that determined in our own studies was noted in soybean cake by Ganzer et al. [37]. Different fat content in protein raw materials was due to the technological processes used. The level of crude fat in soybean meal and in extruded full-fat soybean was close to that determined by Milczarek et al. [6]. Świątkiewicz et al. [38] showed a similar amount (216 g·kg^−1^) of fat in extruded full-fat soybean but a higher amount (88 g·kg^−1^) in soybean expeller cake. According to Niwińska et al. [39], the protein product derived from full-fat non-GM soybean seeds subject to pressure–thermal processing contains a lot of protein and fat as the source of energy and a low amount of anti-nutrients.

The content of anti-nutrients in soybean products depends on the soybean cultivar and refining procedures [6,38,40,41,42]. As regards trypsin inhibitors, it was demonstrated that the amount of these compounds is reduced to the greatest extent by heating [11,20,38,39]. The amount of trypsin inhibitors determined in soybean meal and in extruded full-fat soybean in our own studies corroborated the results of Milczarek et al. [6]. Similarly, Świątkiewicz et al. [38], analysing the content of trypsin inhibitors in soybean products, found 9–11 mg·g^−1^ in extruded full-fat soybean, 6–8 mg·g^−1^ in soybean press cake and 2.8 mg·g^−1^ in standard GM soybean meal. Compared to our own results, Wu et al. [11] determined more trypsin inhibitors in soybean meal.

The content of tannins in soybean meal and in extruded full-fat soybean was close to results obtained by Milczarek et al. [6] who—analysing the content of tannins in the evaluated protein feeds—noted that not only temperature but also humidity and pressure considerably reduce the level of tannins since extruded full-fat soybean contained nearly three times fewer tannins than soybean meal and more than 2.5 times less than raw soybean.

Seeking an alternative method for producing GM soybean meal, the efficiency of extruded full-fat soybean seeds and soybean expeller cake in broiler chicken nutrition was evaluated [6,18,19,20,21,22,43]. In the conducted studies, complete replacement of soybean meal with soybean expeller cake or extruded full-fat soybean allowed increasing bodyweight but feed conversion was better in the group of birds fed with feed rations containing soybean expeller cake. Similarly, Jahanian and Rasouli [42]—replacing all soybean meal with extruded full-fat soybean—found significantly higher weight gain and feed consumption in 5-week-old chickens but the FCR was lower. In turn, Milczarek et al. [6] showed that introducing extruded full-fat soybean into feed rations as a partial substitute of soybean meal (30% in starter; 50% in grower and finisher rations) allowed achieving a similar bodyweight of chickens on Day 42 of rearing (2645 vs. 2564 g) at a similar feed consumption level. In contrast, Zhaleh et al. [44], having added 7.5 or 15% extruded full-fat soybean into feed rations for broiler chickens, found a significant reduction in bodyweight and feed consumption for birds fed with diets in which the level of extruded full-fat soybean was higher, but only in the period from Day 1 to Day 10 of their lives. Foltyn et al. [43], using 4, 8, 12 and 16% extruded full-fat soybean seeds in broiler chicken feed rations, noted a linear decrease in bodyweight (2442.5–2093.1 g) and an increase in feed consumption (1.69–1.90 kg) along with increasing the level of the tested soybean product.

In own studies, soybean expeller cake replacing soybean meal in feed rations for broiler chickens allowed a significant increase in the bodyweight of birds with no effect on feed conversion. In turn, Śliwa and Brzóska [7], analysing the effect of replacing commercial soybean meal with extruded soybean expeller cake from non-GM seeds at an average amount of 10, 18 and 40% of the feed ration, showed that as the share of expeller cake increased in the diet of birds, their bodyweight decreased and the feed conversion rate increased. When the soybean meal was completely eliminated by using the expeller cake, the end bodyweight significantly decreased (7.7%) and the feed conversion rate increased (5.5%). In turn, Powell et al. [19] did not note any significant difference in the carcass weight of slaughtered birds (2.77 vs. 2.75 kg) and in feed conversion rate (0.551 vs. 0.557 g·g^−1^) after introducing expeller-extruded soybean meal as the only source of protein. Similarly, Ganzer et al. [37] did not demonstrate any effect of 10 or 20% soybean cake added to feed rations for ISA J-275 and Ross 308 broiler chickens on bodyweight and feed conversion rate.

The dressing percentage and carcass quality of broiler chickens are affected by many factors, both genetic and environmental, including diet components, their physical form, and the age at which chickens are slaughtered [6,18,19]. The absence of an effect of extruded full-fat soybean used in broiler feed rations on dressing percentage corroborates the results obtained by Subuh et al. [18] and by Jahanian and Rasouli [42]. Subuh et al. [18] demonstrated that extruded soybean seeds can partially or completely replace soybean meal (25/75, 50/50 and 0/100) with no effect on dressing percentage. Similarly, Milczarek et al. [6] found that extruded full-fat soybean added to feed rations allowed obtaining an identical dressing percentage of chickens to that of birds fed with rations containing commercial soybean meal. Śliwa and Brzóska [7], having introduced 10, 18 and 40% of soybean expeller cake into broiler chicken feed rations, did not note any significant effect on dressing percentage.

The share of breast muscles decreased after complete replacement of soybean meal with extruded soybean seeds in feed rations for broiler chickens, which coincides with the results of Powell et al. [19] and of Śliwa and Brzóska [7]. Śliwa and Brzóska [7], using extruded soybean expeller cake in feed rations in the amount of 10, 18 and 40% instead of soybean meal, noted a linear decrease in the share of breast muscles. Extruded full-fat soybean into broiler chicken feed rations significantly increased the fattening grade of the carcasses, while Mirghelenj et al. [21] and Śliwa and Brzóska [7] found a linear decrease in the share of fat reserves along with increased use of extruded full-fat soybean seeds in chicken diets. In turn, Milczarek et al. [6] showed that the type of feed rations used had no significant effect on the share of skin with subcutaneous fat, but it significantly differentiated the share of abdominal fat in the carcasses. Differentiations of obtained results in the above-mentioned experiments could be a using different share of soybean products.

The results of own studies referring to the share of giblets in the bodyweight of birds did not correspond with the findings of other authors [6,7,45]. In studies carried out by Milczarek et al. [6], despite the absence of significant intergroup differences in the share of gizzard, liver and heart, their higher share was observed especially in the group of chickens fed with rations containing raw soybean. Similarly, Pacheco et al. [45], evaluating the effect of feed rations containing extruded soybean seeds, did not show a significant effect of such seeds on the gizzard weight percentage in chicken carcasses. In turn, Śliwa and Brzóska [7] introduced on average 10, 18 and 40% of soybean expeller cake into broiler chicken feed rations but found no effect of it on the share of giblets (heart, liver and gizzard).

Adequate nutrition and proper rearing conditions make it possible to use the genetic potential of broiler chickens and positively affect the quality of meat [46]. Many authors [47,48,49] claim that meat quality is shaped by its sensory traits, hygienic and toxicological traits, physico–chemical parameters and technological characteristics. In our own studies, the analysis of muscle proximate composition revealed significant differences in the content of crude fat. The use of soybean expeller cake in starter and grower rations led to a decrease in the amount of this ingredient both in breast and leg muscles. In turn, Śliwa and Brzóska [7] did not find any significant differences in the chemical composition of muscles, except a significantly higher content of fat in the leg muscles of broiler chickens fed with rations containing 18 or 40% of soybean expeller cake. Similarly, Milczarek and Osek [32] did not demonstrate any effect of using extruded full-fat soybean in diets fed to slaughtered chickens on the proximate composition of breast muscles.

The dietary value of poultry meat is due to its proximate composition and share of fatty acids [32,50,51]. From the point of view of human nutrition, the most notable is the content and ratio of n-6 polyunsaturated fatty acids (PUFA) to n-3 PUFAs. A typical diet in Poland is deficient in n-3 PUFAs, so the ratio of PUFA n-6 to n-3 is 10-30:1, instead of 4:1 as recommended by WHO. A significant increase in the share of linoleic acid (C18:2) and linolenic acid (C18:3) in the muscles of chickens receiving feed rations with extruded full-fat soybean coincides with the results of Milczarek and Osek [32]. The lowest amount of SFA (22.40% and 21.17%) and simultaneously the highest level of PUFA (43.48% and 45.45%) was found in the breast and leg muscles of chickens fed with rations containing extruded full-fat soybean in comparison to chickens from SBM and SEC groups.

The analysis of meat quality includes both its chemical composition and physical traits. A very important characteristic of meat is its reaction decreasing after slaughter due to the increased content of lactic acid in muscles. Many authors [52,53,54] point to a relationship between the technological quality of meat and the rate of decrease in the end pH. If pH drops rapidly, the PSE (pale, soft, exudative) defect can occur in meat. On the other hand, a high post-mortem pH will result in DFD (dark, firm, dry) meat. Both types of meat are undesirable in terms of technological suitability. The optimum pH_24_ of broilers should oscillate around 5.35–6.10, which implies that the evaluated muscles should be classified as normal meat. The results are close to those reported by Rycielska et al. [55]. In turn, Milczarek and Osek [32]—evaluating the effect of including various protein raw materials in feed rations—obtained slightly lower (5.64) pH_24_ of the breast muscles of chickens fed with rations containing extruded full-fat soybean. Probably, the differentiation of fatty acids profile of muscles can be a consequence of using soybean products (SBM, EFS, SEC, soybean oil) in the chicken rations, which originated from different sources.

Another important physical property of meat is its water holding capacity (WHC), which determines the technological suitability of meat for processing. In the opinion of Orkusz [48], WHC can have a positive effect on the meat’s juiciness, shelf life, colour and texture. Water holding capacity is linked to meat reaction as its lowest value is at the isoelectric point of muscle proteins (pH range 5.1–5.3). The further the pH from the isoelectric point, the higher the water holding capacity of muscle proteins is, which leads to increased thermal drip and drip loss. In turn, increased meat juice drip in the packaging increases microbiological contamination and susceptibility to drying out. In our own studies, the breast and thigh muscles of chickens fed with rations containing soybean expeller cake had a higher water holding capacity in comparison to the muscles of birds from other groups (*p* < 0.05). Milczarek and Osek [32] showed that extruded full-fat soybean used as a partial substitute of soybean meal in broiler chicken feed rations had no effect on the water holding capacity of breast muscles. In turn, Zhang et al. [10], using diets containing non-GM and GM soybean meal, did not note their effect on the water holding capacity and thermal drip of broiler chicken muscles.

Meat colour is an important attribute taken into account by buyers, and an important element of evaluating meat dishes during their consumption [56]. According to Zdanowska-Sąsiadek et al. [46], meat of a darker colour resulting from an increased share of oxidised myoglobin is less desired by consumers. As reported by Zdanowska-Sąsiadek et al. [46], Milan and Klaus [54] and Milan et al. [57], meat with a higher pH is darker, which was corroborated by our own studies.

Milczarek and Osek [32] also noted no effect of using extruded full-fat soybean in broiler chicken feed rations. Similarly, Zhang et al. [10], feeding diets containing non-GM and GM soybean meal to chickens, did not observe any impact of the nutrition scheme on the L*, a* and b* parameters of muscles.

The sensory quality of poultry meat is determined by: colour, palatability, tenderness and juiciness. Meat palatability is generated by its flavour and smell and depends on the composition and fatty acids profile of intramuscular fat, the share of connective tissue and thermal processing temperature and type [58]. According to Szkucik et al. [59], palatability is less intense in the breast muscle than in the thigh muscle. Meat juiciness is determined by the content of intramuscular fat, water holding capacity, tenderness, and thermal processing type [47,58]. Replacing soybean meal with soybean expeller cake or extruded full-fat soybean led to a significant modification in the sensory traits of the evaluated muscles. In turn, Milczarek and Osek [32] did not show any impact of replacing soybean meals with extruded full-fat soybean in feed rations for broiler chickens on the sensory qualities of breast muscles.

## 5. Conclusions

The study provides grounds for recommending non-GM soybean expeller cake as a complete substitute for GM soybean meals in broiler chicken nutrition since it generated the best rearing effects and carcass composition as well as physical and sensory properties of muscles. In contrast, from the point of view of human nutrition, extruded full-fat non-GM soybean instead of soybean meals should be recommended for the feed rations of birds as their muscles featured the best fatty acids profile, compared with SBM and SEC groups.

## Figures and Tables

**Table 1:** Chemical composition of soybean feeds.

Item	Soybean Meal GM	Soybean Expeller Cake Non-GM	Extruded Full-Fat Soybean Non-GM
Basal nutrients (g·kg^−1^)			
dry matter	894.6	940.4	939.8
crude ash	70.3	60.0	51.6
crude protein	452.0	443.6	349.5
crude fat	20.7	55.7	218.0
crude fibre	66.0	59.0	61.8
N-free extractives	285.6	322.1	258.9
Gross energy (kcal·kg^−1^)	2868	4730	5420
Anti-nutritional factors (g·kg^−1^)			
trypsin inhibitors	1.40	5.90	8.88
tannins	14.9	2.90	5.08

**Table 2:** Composition and nutritive value of Starter and Grower rations.

Item	Starter			Grower		
	SBM	SEC	EFS	SBM	SEC	EFS
Raw materials and feed additives						
Wheat	54.78	55.93	54.00	59.99	61.24	57.11
Soybean meal	35.00	-	-	29.00	-	-
Soybean press cake	-	35.30	-	-	29.00	-
Extruded full-fat soybean	-	-	41.94	-	-	39.00
Soybean oil	6.00	4.50	-	6.80	5.50	-
Limestone	1.05	1.09	1.26	1.13	1.16	1.32
NaCl	0.35	0.37	0.36	0.37	0.38	0.375
2-Ca phosphate	1.90	1.95	1.60	1.73	1.78	1.40
Premix *	0.50	0.50	0.50	0.50	0.50	0.50
L-lysine	0.16	0.11	0.11	0.23	0.20	0.09
DL-methionine	0.26	0.25	0.23	0.25	0.24	0.21
Calculated nutrients per 1 kg of rations:						
ME (MJ)	12.5	12.5	13.1	12.9	12.9	13.1
crude protein (g)	223	223	211	203	202	204
crude fat (g)	76.6	74.2	100	84.2	81.6	94.7
lysine (g)	12.4	12.4	12.5	11.8	11.8	11.8
methionine (g)	5.64	5.69	5.65	5.30	5.31	5.35
threonine (g)	7.67	7.83	8.00	6.85	6.95	7.67
tryptophan (g)	2.71	2.70	2.92	2.43	2.42	2.81
Ca total (g)	9.55	9.57	9.57	9.32	9.34	9.32
P available (g)	4.37	4.40	4.43	4.05	4.08	4.08
Na (g)	1.56	1.57	1.58	1.62	1.60	1.62

SBM—soybean meal, SEC—soybean expeller cake non-GM, EFS—extruded full-fat soybean non-GM; * 1 kg starter/grower premix contained: vitamin A—2,400,000/2,000,000 IU; D_3_—900,000/800,000 IU; E 9000/7000 IU; K—700/600 mg; B_1_—500/360 mg; B_2_—1200/1000 mg; B_6_—800/700 mg; B_12_ 6000/2600 µg; PP—8000/6000 mg; pantotenian calcium—2600 mg; B_9_ –300/200 mg; H—50,000/40,000 µg; B_4_—70,000/70,000 mg; microelements: Cu—3500/3000 mg; Fe—15,000/12,000 mg; J—350/300 mg; Mn—20,000/18,000 mg; Zn—20,000/20,000 mg; Se—55/90 mg; antioxidant.

**Table 3:** Rearing results of broiler chickens.

Item	Group			SEM	*p*-Value
	SBM	SEC	EFS		
Bodyweight (g)					
1 day	42.9	43.0	43.0	0.096	0.682
21 day	721 ^b^	760 ^a^	715 ^b^	7.42	<0.05
42 day	2294 ^b^	2400 ^a^	2361 ^a^	13.5	<0.05
Bodyweight gain (g)					
1–21 days	678 ^b^	717 ^a^	678 ^b^	7.42	<0.05
22–42 days	1573 ^b^	1639 ^a^	1646 ^a^	12.61	<0.05
1–42 days	2251 ^b^	2357 ^a^	2318 ^a^	13.48	<0.05
Feed conversion ratio (kg)					
1–21 days	1.59 ^b^	1.52 ^c^	1.66 ^a^	0.017	<0.05
22–42 days	1.75 ^a^	1.67 ^b^	1.83 ^a^	0.020	<0.05
1–42 days	1.69 ^b^	1.68 ^b^	1.76 ^a^	0.012	<0.05

SBM—soybean meal, SEC—soybean expeller cake non-GM, EFS—extruded full-fat soybean non-GM, SEM—standard error of mean; ^a,b,c^—means with different superscripts within a row are significantly different at *p* ≤ 0.05.

**Table 4:** Slaughter value of chickens.

Item	Group			SEM	*p*-Value
	SBM	SEC	EFS		
Bodyweight before slaughter (g)	2284 ^b^	2399 ^a^	2352 ^a^	12.98	<0.05
Cold carcass weight (g)	1835	1954	1878	27.72	0.212
Dressing percentage (%)	80.3	81.4	79.8	0.305	0.072
Share in cold carcass (%)					
Muscles total	48.6 ^a^	49.9 ^a^	46.4 ^b^	0.439	<0.05
including:					
breast	29.4 ^a,b^	30.9 ^a^	28.0 ^b^	0.365	<0.05
thigh	11.4	11.2	10.8	0.165	0.396
drumstick	7.90	7.90	7.60	0.104	0.356
Abdominal fat	0.711 ^b^	0.712 ^b^	1.03 ^a^	0.049	<0.05
Skin with subcutaneous fat	7.96 ^b^	8.49 ^b^	9.80 ^a^	0.233	<0.05
Share in bodyweight (%)					
Giblets total	3.34 ^a^	2.72 ^b^	3.10 ^a^	0.065	<0.05
including:					
heart	0.421 ^a,b^	0.391 ^b^	0.452 ^a^	0.037	<0.05
liver	1.69 ^a^	1.45 ^b^	1.72 ^a^	0.192	<0.05
gizzard	1.25 ^a^	0.88 ^b^	0.93 ^b^	0.039	<0.05

SBM—soybean meal, SEC—soybean expeller cake non-GM, EFS—extruded full-fat soybean non-GM, SEM—standard error of mean; ^a,b^—means with different superscripts within a row are significantly different at *p* ≤ 0.05.

**Table 5:** Basal nutrient content (g·100 g^–1^) of muscles.

Item	Groups			SEM	*p*-Value
	SBM	SEC	EFS		
Breast					
dry matter	25.08 ^b^	25.29 ^a,b^	25.68 ^a^	0.103	<0.05
crude ash	1.20	1.20	1.20	0.004	0.907
crude protein	22.15	22.37	22.19	0.091	0.609
crude fat	1.26 ^a,b^	1.10 ^b^	1.41 ^a^	0.042	<0.05
Leg					
dry matter	25.56	25.41	25.73	0.141	0.689
crude ash	1.10	1.06	1.07	0.010	0.221
crude protein	19.97	19.83	19.60	0.111	0.408
crude fat	4.26 ^a,b^	3.87 ^b^	4.48 ^a^	0.106	<0.05

SBM—soybean meal, SEC—soybean expeller cake non-GM, EFS—extruded full-fat soybean non-GM, SEM—standard error of mean; ^a,b^—means with different superscripts within a row are significantly different at *p* < 0.05.

**Table 6:** Fatty acids profile (% of total fatty acids) in muscles.

Fatty Acids	Groups			SEM	*p*-Value
	SBM	SEC	EFS		
Breast muscles					
C14:0	0.145	0.160	0.118	0.010	0.083
C16:0	20.9 ^a^	21 ^a^	17.1 ^b^	0.607	<0.05
C16:1	2.45 ^a^	2.46 ^a^	1.52 ^b^	0.157	<0.05
C17:0	0.188 ^a^	0.185 ^a^	0.155 ^b^	0.006	<0.05
C18:0	5.81 ^a^	5.96 ^a^	4.98 ^b^	0.183	<0.05
C18:1	34 ^a^	34.4 ^a^	32.3 ^b^	0.302	<0.05
C18:2 n-6	33.5 ^b^	32.5 ^b^	40.4 ^a^	1.19	<0.05
C18:3 n-3	1.67 ^b^	1.98 ^a,b^	2.28 ^a^	0.099	<0.05
C20:0	0.138 ^a^	0.118 ^a^	0.020 ^b^	0.014	<0.05
C20:1	0.100	0.115	0.069	0.012	0.134
C20:2	0.095	0.113	0.093	0.006	0.236
C20:3 n-3	0.060	0.063	0.073	0.004	0.412
C20:4 n-6	0.663	0.663	0.605	0.036	0.695
SFA	27.22 ^a^	27.40 ^a^	22.40 ^b^	0.781	<0.05
UFA	72.63 ^b^	72.42 ^b^	77.42 ^a^	0.779	<0.05
MUFA	36.63 ^a^	37.15 ^a^	33.94 ^b^	0.627	<0.05
PUFA	36.00 ^b^	35.27 ^b^	43.48 ^a^	1.25	<0.05
n-6:n-3	20.15 ^a^	16.35 ^b^	17.82 ^a,b^	0.532	<0.05
DFA = (UFA + C18:0)	78.43 ^b^	78.38 ^b^	82.40 ^a^	0.627	<0.05
OFA = (C14:0 + C16:0)	21.08 ^a^	21.13 ^a^	17.22 ^b^	0.612	<0.05
Leg muscles					
C14:0	0.150 ^a^	0.125 ^a^	0.098 ^b^	0.007	<0.05
C16:0	22.4 ^a^	21.8 ^a^	16.8 ^b^	0.697	<0.05
C16:1	2.61 ^b^	3.12 ^a^	1.59 ^c^	0.190	<0.05
C17:0	0.165 ^a,b^	0.145 ^b^	0.210 ^a^	0.011	<0.05
C18:0	5.15 ^a^	4.74 ^a^	3.98 ^b^	0.156	<0.05
C18:1	36.1 ^a^	35.2 ^a^	31.5 ^b^	0.585	<0.05
C18:2 n-6	31.2 ^b^	32.6 ^b^	43 ^a^	1.447	<0.05
C18:3 n-3	1.35 ^c^	1.59 ^b^	2.24 ^a^	0.107	<0.05
C20:0	0.115	0.115	0.113	0.007	0.988
C20:1	0.090	0.065	0.060	0.006	0.053
C20:2	0.135 ^a^	0.035 ^b^	0.030 ^b^	0.014	<0.05
C20:3 n-3	0.043 ^a^	0.013 ^c^	0.030 ^b^	0.004	<0.05
C20:4 n-6	0.198	0.163	0.193	0.011	0.398
SFA	27.99 ^a^	26.89 ^a^	21.17 ^b^	0.827	<0.05
UFA	71.84 ^b^	72.87 ^b^	78.67 ^a^	0.832	<0.05
MUFA	38.90 ^a^	38.45 ^a^	33.21 ^b^	0.753	<0.05
PUFA	32.95 ^b^	34.42 ^b^	45.45 ^a^	1.539	<0.05
n-6:n-3	22.64	20.52	19.20	0.729	0.095
DFA = (UFA + C18:0)	76.99 ^b^	77.91 ^b^	82.64 ^a^	0.695	<0.05
OFA = (C14:0 + C16:0)	22.56 ^a^	21.97 ^a^	16.87 ^b^	0.702	<0.05

SBM—soybean meal, SEC—soybean expeller cake non-GM, EFS—extruded full-fat soybean non-GM, SEM—standard error of mean; SFA—saturated fatty acids, UFA—unsaturated fatty acids, MUFA—monounsaturated fatty acids, PUFA—polyunsaturated fatty acids, DFA—neutral and hypocholesterolemic fatty acids, OFA—hypercholesterolemic fatty acids, SEM—standard error of mean; ^a,b,c^—means with different superscripts within a row are significantly different at *p* < 0.05.

**Table 7:** Physical properties of muscles.

Item	Groups			SEM	*p*-Value
	SBM	SEC	EFS		
Breast					
pH_15_	5.89	5.91	5.92	0.032	0.975
pH_24_	5.69	5.70	5.72	0.013	0.081
L*	52.2	53.3	53.2	0.603	0.714
a*	2.44 ^b^	4.43 ^a^	2.03 ^b^	0.350	<0.05
b*	0.71 ^b^	1.16 ^a^	0.82 ^b^	0.194	<0.05
C* = [(a*)^2^ + (b*)^2^]^0.5^	2.63 ^b^	4.72 ^a^	2.51 ^b^	0.344	<0.05
H = log (b*/a*)	0.344 ^b^	0.320 ^b^	0.605 ^a^	0.126	<0.05
WHC (%)	13.1 ^a^	9.8 ^b^	12.0 ^a^	0.691	<0.05
Thigh					
pH_15_	5.88	5.90	5.89	0.019	0.647
pH_24_	5.95 ^a^	5.88 ^a,b^	5.81 ^b^	0.022	<0.05
L*	48.1	50.2	48.9	0.682	0.443
a*	4.30 ^b^	4.51 ^b^	5.44 ^a^	0.376	<0.05
b*	0.696 ^a^	0.881 ^a^	0.388 ^b^	0.257	<0.05
C* = [(a*)^2^ + (b*)^2^]^0.5^	4.72	4.82	5.73	0.339	0.420
H = log (b*/a*)	0.613 ^a^	0.203 ^b^	0.179 ^b^	0.123	<0.05
WHC (%)	8.97 ^a^	5.67 ^b^	9.63 ^a^	0.566	<0.05

SBM—soybean meal, SEC—soybean expeller cake non-GM, EFS—extruded full-fat soybean non-GM, SEM—standard error of mean; L*—lightness, a*—redness, b*—yellowness, C*—chroma, H—hue, WHC—water holding capacity; ^a,b,c^—means with different superscripts within a row are significantly different at *p* < 0.05.

## Data Availability

The data are available on request from the corresponding author.
